# Vaccine and Vaccination: On Field Research

**DOI:** 10.3390/vaccines10071027

**Published:** 2022-06-27

**Authors:** Francesca Gallè, Christian Napoli

**Affiliations:** 1 Department of Movement Sciences and Wellbeing, University of Naples “Parthenope”, Via Medina n. 40, 80133 Naples, Italy; 2 Department of Medical Surgical Sciences and Translational Medicine, “Sapienza” University of Rome, Via di Grottarossa 1035/1039, 00189 Rome, Italy; christian.napoli@uniroma1.it

## 1. Background

Historically, vaccinations have enabled the eradication, elimination, and control of many debilitating diseases. In 2020, during the COVID-19 pandemic, the scientific community started working tirelessly on the development of an appropriate vaccine, highlighting the importance of vaccination as a powerful life-saving weapon in the fight against infectious diseases. However, despite the real danger that infectious diseases pose to health and the proven historical importance of vaccines, informed adherence to vaccination is currently significantly undervalued in common perception. Compulsory immunization plans must be accompanied by effective education and information strategies, paying attention to the spread of data not supported by scientific evidence. In this context, this Special Issue, “Vaccines and Vaccination: On Field Research”, has the main objective of increasing the evidence and observations of the international literature concerning (i) vaccine epidemiology, (ii) vaccine hesitancy and COVID-19 vaccine acceptance, (iii) and immunization strategies aimed at improving vaccination coverage through education and communication campaigns for both health professionals and the general population.

In recent centuries, since their development, vaccinations have enabled the eradication of smallpox, drastically reduced the incidence of debilitating diseases such as polio and measles, and even prevented the onset of certain noncommunicable diseases, including liver and cervical cancer, thereby helping to increase the life expectancy of the world’s population [1]. Recently, the coronavirus 2019 (COVID-19) pandemic posed a humanitarian crisis marked by significant health, psychological, and economic pressures that exposed the vulnerability of institutional, scientific, and health systems worldwide. Seeking a prompt response to the pandemic emergency, in 2020 public health, alongside global health, authorities moved swiftly to coordinate numerous clinical trial projects seeking vaccine candidates against SARS-CoV-2. Besides the development of effective therapies and tools to identify infected individuals, an important part of the scientific community worked tirelessly on the development of an appropriate vaccine [2]. In fact, the COVID-19 pandemic has been a reminder of the importance of vaccination as a powerful life-saving weapon in the fight against infectious diseases.

However, despite the real danger that infectious diseases pose to health and the proven historical importance of vaccines, informed adherence to vaccination is currently significantly undervalued in common perception [3]. In this context, altered perceptions of health risks have a significant impact on the health decisions taken by the population. In fact, the discrepancy between the real danger and the perceived risk can lead to inappropriate behavior that does not comply with the public health measures recommended for both the general population and at-risk cohorts, such as healthcare workers in both hospital and community settings [3,4,5]. Additionally, where sufficient levels of knowledge about COVID-19 characteristics and prevention are demonstrated, effective warnings and messages issued by public health communicators seem to be still necessary [6]. Indeed, since people no longer experience those infectious diseases that have been reduced or eliminated through vaccination, many individuals do not perceive the need to immunize themselves and their children, thus increasing their possibility of contracting natural diseases and developing related sequelae [7]. In a world characterized by global movements of populations, this problem is even more important [8,9].

In particular, the widely known phenomenon of vaccine hesitancy, described in 2019 as one of the ten greatest threats to global health by the World Health Organization, has also affected COVID-19 vaccination, hindering the attainment of the desired vaccination coverage, sometimes even in hospital settings. Indeed, in the health and social care setting, healthcare workers (HCWs) are not exempt from the phenomenon of vaccine hesitancy, which is worrying considering that the lack of awareness of the importance of vaccination in this cohort of individuals conflicts with the principles on which the healthcare profession’s mission is founded [10].

Different studies have demonstrated a high level of acceptance towards COVID-19 vaccination and a good level of knowledge regarding vaccine characteristics in the general population. Moreover, knowledge and vaccine acceptance were found to be correlated [11]. After the occurrence of COVID-19 vaccine complications, peoples’ perceptions of vaccine negative health consequences increased. At the same time, surprisingly, the acceptance of COVID-19 vaccination also increased significantly, probably due to the fact that at the beginning of the COVID vaccination campaign people were more worried about COVID-19 than about the possible adverse effects of vaccination [11]. However, later, after the mandatory implementation of the green pass, a lower vaccine acceptance was registered, suggesting that compulsory immunization plans must be accompanied by effective education and information strategies, paying attention to the spread of data not supported by scientific evidence [12]. In this context, healthcare personnel play a crucial role, since they can tailor communication to those categories who were shown to be more hesitant.

Moreover, in a landscape in which a sense of suspicion and mistrust towards vaccinations and health institutions has grown, the traditional counter tools have been shown to be ineffective. This is further amplified today by the ease with which information can be spread, thanks to new media and social networks [10,13].

In particular, during the current pandemic, continuous media exposure to a huge volume of seemingly conflicting news (an infodemic) made it difficult for inexperienced users to find reliable sources of information [14].

Given the potential of technology in information-seeking processes, the use of online channels by healthcare institutions could be a valuable tool for divulging medical and scientific knowledge to citizens [14]. International and national health authorities as well as agencies must collaborate in adopting these measures in order to guide the population towards consciously chosen decisions.

## 2. Conclusions

This Special Issue, “Vaccines and Vaccination: On Field Research”, has the main objective of increasing the evidence and observations of the international literature concerning (i) vaccine epidemiology, (ii) the phenomenon of vaccine hesitancy and COVID-19 vaccine acceptance, and (iii) the adoption of vaccination strategies in order to improve vaccination coverage of the population through health activities, education, and communication campaigns for both the general public and health professionals. To this end, original articles, systematic reviews or meta-analyses, short communications, commentaries, or other types of articles on vaccines administered in all cohorts of the population (infants, adolescents, adults, elderly people, and at-risk populations) as well as strategies adopted to promote vaccination adherence among these categories are welcomed and encouraged for this Special Issue.
